# Medication Adherence and Cardiometabolic Control Indicators Among American Indian Adults Receiving Tribal Health Services: Protocol for a Longitudinal Electronic Health Records Study

**DOI:** 10.2196/39193

**Published:** 2022-10-24

**Authors:** Lisa Scarton, Tarah Nelson, Yingwei Yao, Richard Segal, William T Donahoo, R Turner Goins, Ashley DeVaughan-Circles, Spero M Manson, Diana J Wilkie

**Affiliations:** 1 College of Nursing University of Florida Gainesville, FL United States; 2 College of Pharmacy University of Florida Gainesville, FL United States; 3 College of Medicine University of Florida Gainesville, FL United States; 4 College of Health and Human Sciences Western Carolina University Cullowhee, NC United States; 5 Choctaw Nation of Oklahoma Talihina, OK United States; 6 Colorado School of Public Health University of Colorado Anschutz Medical Campus Aurora, CO United States

**Keywords:** medication adherence, American Indian, type 2 diabetes

## Abstract

**Background:**

American Indian adults have the highest prevalence of type 2 diabetes (T2D) in any racial or ethnic group and experience high rates of comorbidities. Uncontrolled cardiometabolic risk factors—insulin resistance, resulting in impaired glucose tolerance, dyslipidemia, and hypertension—increase the risk of mortality. Mortality is significantly reduced by glucose- and lipid-lowering and antihypertensive medication adherence. Medication adherence is low among American Indian adults living in non–Indian Health Service health care settings. Virtually nothing is known about the nature and extent of medication adherence among reservation-dwelling American Indian adults who primarily receive their medications without cost from Indian Health Service or tribal facilities. Electronic health records (EHRs) offer a rich but underused data source regarding medication adherence and its potential to predict cardiometabolic control indicators (C-MCIs). With the support of the Choctaw Nation of Oklahoma (CNO), we address this oversight by using EHR data generated by this large, state-of-the-art tribal health care system to investigate C-MCIs.

**Objective:**

Our specific aims are to determine, using 2018 EHR data, the bivariate relationships between medication adherence and C-MCIs, demographics, and comorbidities and each C-MCI and demographics and comorbidities; develop machine learning models for predicting future C-MCIs from the previous year’s medication adherence, demographics, comorbidities, and common laboratory tests; and identify facilitators of and barriers to medication adherence within the context of social determinants of health (SDOH), EHR-derived medication adherence, and C-MCIs.

**Methods:**

Drawing on the tribe’s EHR (2018-2021) data for CNO patients with T2D, we will characterize the relationships among medication adherence (to glucose- and lipid-lowering and antihypertensive drugs) and C-MCIs (hemoglobin A1c ≤7%, low-density lipoprotein cholesterol <100 mg/dL, and systolic blood pressure <130 mm Hg); patient demographics (eg, age, sex, SDOH, and residence location); and comorbidities (eg, BMI ≥30, cardiovascular disease, and chronic kidney disease). We will also characterize the association of each C-MCI with demographics and comorbidities. Prescription and pharmacy refill data will be used to calculate the proportion of days covered with medications, a typical measure of medication adherence. Using machine learning techniques, we will develop prediction models for future (2019-2021) C-MCIs based on medication adherence, patient demographics, comorbidities, and common laboratory tests (eg, lipid panel) from the previous year. Finally, key informant interviews (N=90) will explore facilitators of and barriers to medication adherence within the context of local SDOH.

**Results:**

Funding was obtained in early 2022. The University of Florida and CNO approved the institutional review board protocols and executed the data use agreements. Data extraction is in process. We expect to obtain results from aims 1 and 2 in 2024.

**Conclusions:**

Our findings will yield insights into improving medication adherence and C-MCIs among American Indian adults, consistent with CNO’s State of the Nation’s Health Report 2017 goal of reducing T2D and its complications.

**International Registered Report Identifier (IRRID):**

PRR1-10.2196/39193

## Introduction

### Background

American Indian adults have the highest prevalence of type 2 diabetes (T2D) in any racial and ethnic group and have high rates of comorbidities [1]. Uncontrolled cardiometabolic risk factors—insulin resistance, resulting in impaired glucose tolerance, dyslipidemia, and hypertension (HTN)—increase the risk of mortality. Mortality is significantly reduced by glucose- and lipid-lowering and antihypertensive medication adherence [2,3]. Medication adherence is low among American Indian adults living in non–Indian Health Service (IHS) health care settings [4], whereas virtually nothing is known about the nature and extent of medication adherence among reservation-dwelling adults who primarily receive their medications without cost from IHS or tribal facilities. Electronic health record (EHR) systems at these facilities offer a rich but underused data source about medication adherence and its potential to predict cardiometabolic control indicators (C-MCIs), for example, hemoglobin A1c (HbA1c), low-density lipoprotein cholesterol (LDL-C), and systolic blood pressure (SBP). We will address this oversight by using EHR data from a large tribal health care system to investigate medication adherence and C-MCIs to inform future interventions.

Prior evidence of medication adherence among reservation-dwelling adults includes 2 small studies with extremely low medication adherence [5,6] and another study across 5 American Indian tribes (n=166), in which 72% had low to moderate medication adherence [7]. American Indian adults with T2D who received care in a non-IHS setting were significantly less likely to adhere to oral T2D medication than non-Hispanic White adults [4]. Among Medicare enrollees with T2D, *older age*, being *female*, and cost of copays were significant predictors of nonadherence [8,9]. Other knowledge about C-MCIs in the American Indian population is derived largely from the Strong Heart Study (SHS), the largest population-based cohort study of American Indian adults [10,11]. Its findings documented that cardiovascular complications are related to *sex, older* *age, T2D, HTN,* obesity (*BMI*>30), and *hyperinsulinemia* [12,13]. However, the SHS did not assess medication adherence, focused only on older adults (45-74 years), and examined cardiovascular risk factors through cross-sectional interviews [11]. Our study builds on the SHS by considering the relationship between medication adherence and C-MCIs (*HbA1c, LDL-C, and SBP*), longitudinally estimating future C-MCIs across a broader age span, and describing facilitators of and barriers to medication adherence. Our preliminary findings suggest that from 43% to 65% of Choctaw Nation of Oklahoma (CNO) adults have above-target C-MCIs and 37% have medication adherence below the 0.8 target for metformin, an oral T2D medication (Scarton, L, unpublished data, September 2022). CNO patients receive most or all of their health care within one tribal system, allowing us to track medication adherence and C-MCIs over a 4-year longitudinal study. The prevalence of T2D and C-MCIs is increasing among young adults and is higher in rural communities such as CNO, whose citizens are affected by high rates of poverty, distant health care, and difficulty transiting tribal lands spread over 11,000 square miles [14-16]. Diabetes distress, depressive symptoms, forgetting to take medications, adverse effects, cost, and knowledge gaps are known barriers to medication adherence [5,17,18], but little is known about medication adherence facilitators, other barriers, and the role of social determinants of health (SDOH) among reservation-dwelling American Indian adults.

Drawing on the tribe’s EHR (2018-2021) data for the (5970 in 2017) CNO patients with T2D, we will characterize the relationships among medication adherence (to glucose- and lipid-lowering and antihypertensive drugs) and *C-MCIs* (HbA1c ≤7%, LDL-C <100 mg/dL, and SBP <130 mm Hg); patient *demographics* (eg, age, sex, SDOH, and residence location); and *comorbidities* (eg, BMI ≥30, cardiovascular disease [CVD], and chronic kidney disease [CKD]). We will also characterize the association of each *C-MCI* with *demographics* and *comorbidities*. Prescription and pharmacy refill data will be used to calculate the proportion of days covered (PDC) with medications, a typical measure of medication adherence [19]. Using machine learning techniques, we will develop prediction models for future (2019-2021) C-MCIs based on *medication adherence, patient demographics, comorbidities, and common laboratory tests* (eg, lipid panel) from the previous year. Such techniques have advantages over traditional algorithms related to CVD outcomes [20-22] but none have been applied to American Indian populations. Finally, key informant interviews (N=90) will explore facilitators of and barriers to medication adherence within the context of local SDOH.

### Study Aims

This study addresses the following 3 aims among American Indian adults with T2D.

#### Aim 1

Using the 2018 EHR data, we will determine the bivariate relationships between *medication adherence* and C-MCIs, demographics, and comorbidities (aim 1a). We expect that adherence will be positively associated with HbA1c ≤7%, LDL-C <100 mg/dL, SBP <130 mm Hg, younger age, male sex, nonrural residence, and BMI <30. We will also determine the relationship between each C-MCI and demographics and comorbidities (aim 1b). We expect decreased C-MCIs in older adults or those with BMI ≥30, higher HbA1c and LDL-C in females, and higher SBP in males.

#### Aim 2

We will develop machine learning models (eg, random forest and nearest neighbors) for predicting future (2019-2021) C-MCIs from the previous year’s medication adherence, demographics, comorbidities, and common laboratory tests. We will compare the models and identify the ones that perform best in predicting at-risk patients. Interpretable models (eg, penalized logistic regression) for both medication adherence and C-MCIs will be developed to identify modifiable factors as potential intervention targets.

#### Aim 3

We will identify facilitators of and barriers to medication adherence within the context of SDOH, EHR-derived medication adherence (PDC), and C-MCIs (at target, above target, and uncontrolled HbA1c).

### Research Strategy and Significance

American Indian adults are >2.5 times as likely to have T2D compared with other racial and ethnic groups [23]. Although African Americans and Hispanic adults have higher rates of T2D than non-Hispanic White adults (12%, 12.5%, and 7.5%, respectively), American Indian adults experience even greater rates between 15% and 50% [23]. Among CNO tribal patients living within CNO tribal boundaries in Oklahoma, approximately 16% have been diagnosed with T2D [24]. T2D can often be prevented or managed by monitoring HbA1c levels, lifestyle changes, and medications. American Indian adults with T2D have poorer glycemic control (higher HbA1c values) compared with their White counterparts, putting American Indian adults at increased risk for developing diabetes-related complications [25] and increased prevalence of above-target C-MCIs, which can lead to high rates of mortality. Targeting HbA1c levels alone will not eliminate these morbidities. Focus is also needed on other C-MCIs (eg, LDL-C and SBP) and adherence to medications that reduce them.

### Conceptual Framework

**Figure 1:**
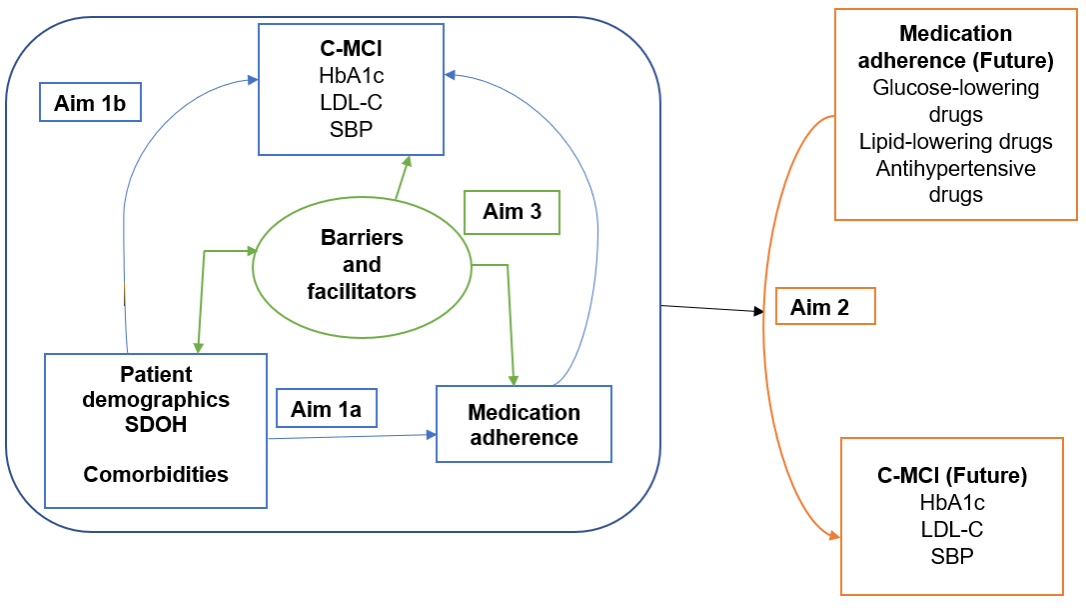
Conceptual framework. C-MCI: cardiometabolic control indicator; HbA1c: hemoglobin A1c; LDL-C: low-density lipoprotein cholesterol; SBP: systolic blood pressure; SDOH: social determinants of health.

Figure 1 illustrates the conceptual framework of this study. This framework is guided by SDOH, which are known to contribute to health disparities and impact T2D health outcomes [26]. SDOH include factors such as socioeconomic status, education, employment, and access to health care. In addition, there is strong scientific evidence regarding how medication adherence affects the control of cardiometabolic conditions, as evidenced by HbA1c, LDL-C, and SBP levels [2,3]. There is also some evidence that adverse SDOH are associated with lower overall medication adherence [27]. For example, long-distance commutes to receive health care and housing instability were associated with lower medication adherence [27]. As shown in Figure 1 and supported by evidence from other populations, the conceptual framework shows the expected relationships to be examined by each of the 3 study aims. Among American Indian adults receiving care from a tribal health care system that provides services and medications without copay, medication adherence is expected to be associated with C-MCIs, as well as the patient demographics, SDOH, and comorbidities (aim 1a) and patient demographics (including residence location); SDOH and comorbidities are expected to be associated with C-MCIs (aim 1b). Patient demographics, SDOH, comorbidities as indicated by common laboratory values and complications, and medication adherence of the previous year will predict C-MCIs of the future year (aim 2). Finally, insights gained from qualitative interviews with a large sample of patients with different levels of C-MCIs and medication adherence (aim 3) will inform future model development and the creation and testing of interventions focused on improving medication adherence and C-MCIs.

## Methods

### Overview

**Figure 2:**
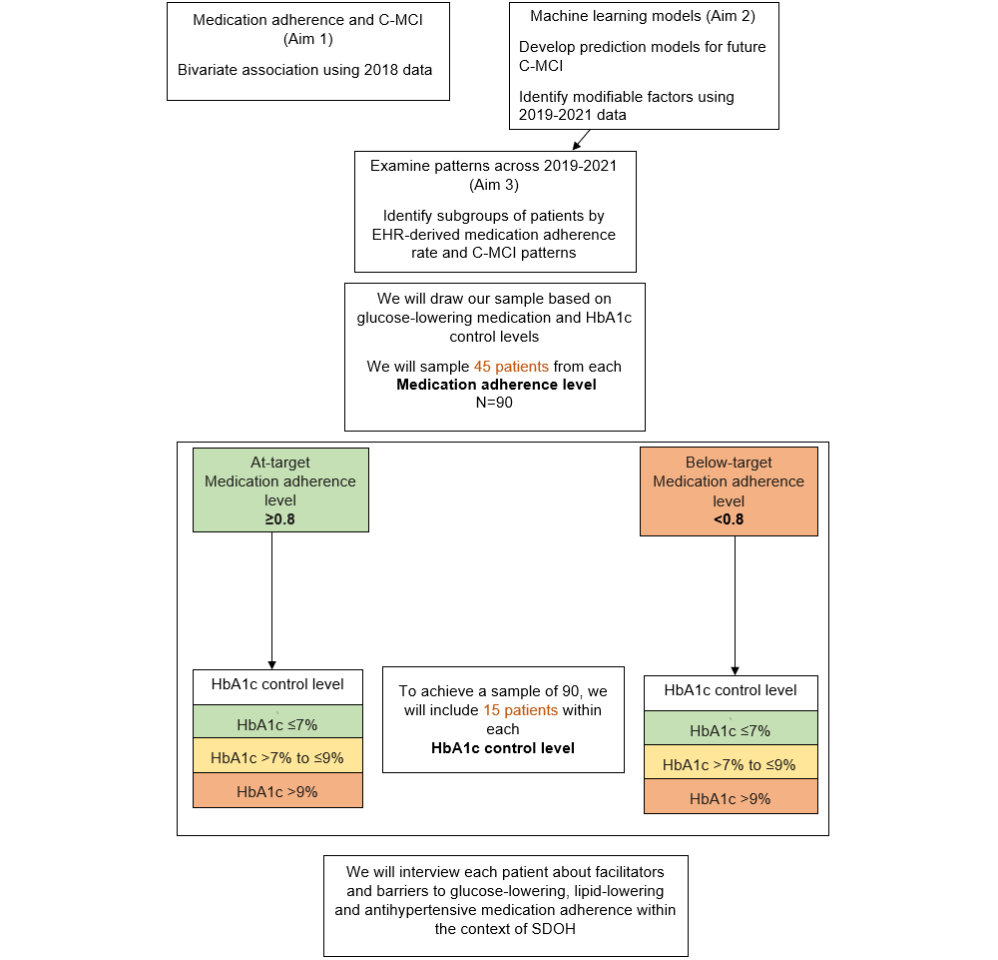
Flow diagram. C-MCI: cardiometabolic control indicator; EHR: electronic health record; HbA1c: hemoglobin A1c; SDOH: social determinants of health.

The Methods section, from research procedures to analysis, are described by aims 1 to 3. Owing to the differences in the samples, we first discuss aims 1 and 2 and then aim 3. The procedure flow diagram (Figure 2) shows the flow from aim 1 to aim 3 as well as the approach for drawing the aim 3 sample.

### Aims 1 and 2

#### Research Procedures

Our study will draw upon existing CNO EHR data. The CNO has a robust, modern integrated EHR data system. These EHR data represent clinical measures as indicators of CNO program outcomes from 2018 to 2021.

The CNO uses an EHR system designed specifically for IHS and tribal health care facilities, the EHR Resource and Patient Management System, which provides CNO access to personal health information and epidemiological data. Information available through this system includes diagnosis of chronic and acute conditions in International Classification of Diseases, Tenth Revision (ICD-10) codes, prescription and dispensing data, information on processes of care procedures and monitoring (eg, blood pressure, screening, and annual health checks), and demographics (eg, age, sex, SDOH, and residence location). CNO pharmacy refill data are linked to EHR data and provide information such as dosage regimen (eg, dose, route of administration, and how to take the medication), date of new medication prescribed, date when picked up, and refill dates. This system is integrated across acute care hospitals, clinics, and other health care facilities (eg, diabetes wellness centers).

All encounters with CNO health system (internal or external) are captured within the EHR unless the patient seeks care outside CNO health system network and pays out of pocket for services. Any patient referred to a specialty clinic outside of CNO will still be captured in the EHR. There may be some patients without office visits or laboratory results within 4 years after the medication fill. The American Diabetes Association guidelines recommend that patients be seen at least every 3 to 6 months until their HbA1c levels are under control. Therefore, we anticipate only a few patients without any follow-up visits. In addition, CNO providers recommend that patients be seen at least every 6 months for diabetes follow-up. Nonetheless, the frequency of patients who fill their medication and the number of patients who do not follow up with their primary care physician during the next 3 years will be very helpful information for designing interventions.

#### Setting

CNO was originally located in the southeastern United States (Alabama, Florida, Mississippi, and Louisiana) until the Choctaw Removal in the early 1830s. The removal, known as the Trail of Tears, split the tribe into what is now 3 federally recognized tribes: CNO, Jena Band of Choctaw Indians, and Mississippi Band of Choctaw Indians. The study will be conducted with CNO, which spreads over 11,000 square miles in 10.5 counties in rural southeastern Oklahoma. CNO, the third largest federally recognized American Indian tribe, has over 200,000 tribal members throughout the United States [28]. The 4 major health challenges associated with CNO have remained the same over the last several years: T2D, HTN, obesity, and CVD [24]. Although CNO has implemented many health promotion programs and is dedicated to the well-being of citizens, SDOH concerns such as poverty, long distance to health clinics, and discrimination continue to persist.

#### Sample Eligibility Criteria

Criteria for *inclusion* in the analysis are tribal members who (1) have at least one health system encounter between January 1, 2018, and December 31, 2021 (office visits, laboratory results, and medication fill), (2) are aged ≥18 years, and (3) have T2D based on ICD-10 codes. We will *exclude* patients who fill their prescriptions outside of CNO and those with end-stage renal disease (ESRD).

#### Sample Power

We estimated the power of the sample based on 5970 patients with T2D in 2017. In this population, 56% (3343/5970) had HbA1c >7%, 43.39% (2057/4741) had an LDL-C level of 100 mg/dL or higher, and 65.31% (3891/5958) had an SBP ≥130 mm Hg. To accommodate the multiple hypotheses to be tested in our aims, we will set the level of significance at a 2-sided α of .002. We assume conservatively that medication adherence, as measured by the PDC, has an SD of 0.2, based on the study by Borne et al [19] as well as our preliminary findings, where PDCs for various medications had an SD of 0.2 or lower. For each of the 3 classes of medications, over 67.82% (4049/5970) of our sample had prescriptions. The proposed sample had sufficient power (80%) to detect a 6.2% difference between the 2 balanced groups in the prevalence of HbA1c >7%, LDL-C ≥100 mg/dL, and SBP ≥130 mm Hg. The power is lower when the groups are unbalanced, but even for a very unbalanced 80/20 split, the proposed sample would have sufficient power (80%) to detect a 7.7% difference in the prevalence of these conditions. The sample will have sufficient power (80%) to detect a 2.5% difference in medication adherence between the 2 balanced groups and a 3.1% difference in an unbalanced 80/20 split.

We will extract the EHR variables listed in Table 1. We will examine medication by class: antihypertensive (eg, angiotensin-converting enzyme inhibitors), glucose-lowering (eg, metformin), and lipid-lowering (eg, statins) measured by PDC based on prescriptions and refills between January 2018 and December 2021.

**Table 1:** Data domain, measures, and clinic visit time points extracted from EHR^a^.

Domain and measure (target value and description)^b^		Collection time points
**Medications, prescriptions, and refills**		
	PDC^c^ glucose-lowering agents	Date of first dispensing the medication in 2018 to the last dispensing in 2021
	PDC lipid-lowering agents	Date of first dispensing the medication in 2018 to the last dispensing in 2021
	PDC antihypertensive agents	Date of first dispensing the medication in 2018 to the last dispensing in 2021
**C-MCI^d^**		
	HbA1c^e^ (≤7%)	All visits in measurement period
	LDL-C^f^ (<100 mg/dL)	All visits in measurement period
	SBP^g^ (<130 mm Hg)	All visits in measurement period
**Patient demographics**		
	Age (date of birth)	As of January 1, 2018
	Sex (male or female)	As of January 1, 2018
	Community of residence (coded)	As of January 1, 2018
**Comorbid conditions**		
	CVD^h^, CKD^i^, and obesity with BMI (≥30)	As of January 1, 2018
**Common laboratory tests**		
	eGFR^j^, lipid panel, CMP^k^, HbA1c, and CBC^l^	All visits in measurement period

^a^EHR: electronic health record.

^b^Values will be collected from patients aged ≥18 years with type 2 diabetes (based on the International Classification of Diseases, Tenth Revision code).

^c^PDC: proportion of days covered.

^d^C-MCI: cardiometabolic control indicators.

^e^HbA1c: hemoglobin A1c values defined according to the American College of Physicians guidelines for 2018.

^f^LDL-C: low-density lipoprotein cholesterol values defined according to the American College of Physicians guidelines for 2018.

^g^SBP: systolic blood pressure values defined according to the American College of Physicians guidelines for 2018.

^h^CVD: cardiovascular disease.

^i^CKD: chronic kidney disease.

^j^eGFR: estimated glomerular filtration rate.

^k^CMP: comprehensive metabolic panel.

^l^CBC: complete blood count.

#### Data Extraction Process

Qualified and experienced CNO program staff members agreed to extract data (January 1, 2018, to December 31, 2021) from CNO EHR database according to a list of variables in our data dictionary. The data will include medication prescriptions and pharmacy refill data from CNO EHR. We chose a 4-year timeframe to ensure adequate capture of pharmacy dispensing data, based on a similar study [29]. The data will be stored on a University of Florida (UF) password-protected file server in a secure environment. Only the research team members will have access to the raw data. Any paper data resulting from the study (such as a printout of part of the electronic file) will be stored in locked file cabinets in a locked office.

#### Measures

Control indicators (HbA1c, LDL-C, and SBP) were measured using labs collected at each visit within the measurement period. The data domains, measures, and clinic visit time points are listed in Table 1.

HbA1c and LDL-C levels were analyzed by 1 of the 9 CNO labs. HbA1c is a reliable test that measures the amount of glucose attached to hemoglobin over the past 3 months. This assay will determine the patient’s metabolic control over a 3-month period [30]. The target HbA1c range is ≤7% for individuals with T2D [31]. LDL-C level is associated with an increased risk of developing heart disease. The target LDL-C range is <100 mg/dL [32]. SBP is obtained in the clinic environment by a licensed health professional or registered nurse and entered into the EHR system. The target SBP range is <130 mm Hg [33].

Medication adherence was measured for medications that were prescribed at least once. Each patient’s adherence was assessed based on the methodology developed by the Pharmacy Quality Alliance (PQA), called PDC. The PDC is widely used by health plan accreditors and in Medicare Part D Drug Plan Star Ratings as a proxy measure for adherence to medications used in the treatment of T2D, HTN, and high blood cholesterol [34]. The PDC is the proportion of calendar days in a period in which a patient had medication on hand to treat their chronic condition. PDC are commonly classified into 2 categories where ≥0.8 is considered adherent and <0.8 not adherent. The threshold of 0.8 for adherence is the level above which medication in these drug classes has a reasonable likelihood of achieving clinical benefit [35], although Lo-Ciganic et al [36] and Tuller et al [37] recommend that PDC be measured continuously among patients with diabetes on hypoglycemic agents. Given these considerations, adherence will be operationalized as a continuous measure of PDC within a range of 0 to 1. We will also report the percentage of samples that were adherent (PDC ≥0.8) and nonadherent (PDC <0.8).

Persons with ESRD are excluded from the PDC measure because adherence to glucose-lowering medications may not be accurately reflected in pharmacy claims data because of frequent dosage and medication adjustments. Patients undergoing peritoneal dialysis may also have glucose-containing dialysate that influence glycemic control, which leads to resultant adjustments to diabetes medications. Furthermore, the PQA recommends that patients on insulin be excluded from the PDC measure because insulin requires titration and frequent dosage adjustments, and despite directions to discard insulin vials after 30 days, many patients continue to use the insulin beyond 30 days [38]. However, as insulin is used by a significant percentage of the study sample and because insulin use is difficult for many individuals, we will calculate the PDC for patients who are dispensed insulin, noting that these data should be interpreted cautiously.

As we have access to the EHRs of enrolled patients, we will also determine primary medication nonadherence (PMN) events, which are recommended by the PQA to assess when a new medication is prescribed for a patient, but the patient does not obtain the medication, or an appropriate alternative, within an acceptable period after it was prescribed [39]. PMN will be reported at the individual patient level based on prescriptions for medications prescribed for the treatment of T2D, HTN, and high blood cholesterol. A PMN will be identified when there is no pharmacy dispensing event that matches the patient and the prescribed drug within 30 days following the prescribing event based on medication information documented by the medical provider in the EHR and within-pharmacy refill data. Medications that are not new will be excluded from the PMN calculation. Thus, prescriptions refilled in the preceding 180 days for the same drug will be excluded [39].

In the study analyses, we will calculate the PDC at the level of the medical condition (ie, T2D, HTN, and high blood cholesterol). For patients prescribed multiple medications for a medical condition, the PQA methodology for multiple medications will be used in the calculations of the PDC. For example, in T2D, where a patient has been prescribed multiple glucose-lowering drugs, participants need coverage with at least one medication for each day of the treatment period. Coverage can include different types of T2D medications throughout the treatment period, as long as all medications are on the target medication list [38].

#### Data Management

Deidentified data will be imported into statistical software R for cleaning and analysis. We will use custom scripts written for this project to rigorously audit the data for validity (ie, conformation to range, data type, set membership, and other constraints), consistency (between different variables and between values of the same variable across time), and completeness (missing data).

#### Data Analysis

##### Aim 1: To Determine the Bivariate Relationships Between (1) Medication Adherence and C-MCIs, Patient Demographics, and Comorbidities and (2) Each C-MCI and Patient Demographics and Comorbidities

We will use descriptive statistics to summarize demographic and clinical variables using the 2018 data. We will calculate medication adherence PDC for glucose- and lipid-lowering and antihypertensive drugs using the pharmacy data previously described. We define above-target C-MCIs as HbA1c >7%, LDL-C ≥100 mg/dL, and SBP ≥130 mm Hg and will use the latest values of these variables in 2018 in this analysis. Regression modeling and likelihood ratio test will be used to examine associations between medication adherence and C-MCI as well as between them and comorbidities and patient demographic variables including age, sex, and residence location.

##### Aim 2: To Develop Machine Learning Models (eg, Random Forest, Nearest Neighbors, and Others) for Predicting Future (2019-2021) C-MCIs from the Previous Year’s Medication Adherence, Patient Demographics, Comorbid Conditions, and Common Laboratory Tests

Unlike traditional regression, machine learning focuses on the prediction performance of the overall model rather than the individual predictors. Through regulation and hyperparameter tuning, it automatically adjusts the model complexity, balancing bias and variance to achieve optimal prediction performance, and does not need to rely on an arbitrary threshold for statistical significance. In addition, machine learning methods such as random forest and nearest neighbor can implicitly accommodate nonlinearity and high-order interactions. In traditional regression analysis, they must be manually prespecified to be included in the model, which is an almost impossible task given the large number of possibilities. Traditional model selection approaches such as stepwise selection also lead to overfitting, resulting in inferior prediction performance.

We will construct machine learning classifiers (random forest, nearest neighbors, and others) to predict future C-MCIs based on the previous year’s medication adherence, patient demographics (eg, age, sex, SDOH, and residence location), comorbidities (eg, CVD, BMI ≥30, and CKD), and common laboratory tests (eg, e-GFR and lipid panel). We will use a random 80/20 partition to reserve 20% (1194/5970) of the patient sample as the test set, with the remaining 80% (4776/5970) as the training and validation set. All modeling will be performed on the training and validation set, including coding of the predictors, model training, model validation, hyperparameter tuning, and model selection. We will use nested cross-validation: the inner (10-fold) cross-validation will be used to choose hyperparameters for a machine learning algorithm, whereas the outer (leave-one-out) cross-validation will be used to evaluate the generalization performance of a model, enabling comparison of different modeling approaches to select the optimal model. The finalized model will be assessed using the reserved test data set to obtain an unbiased estimate of its performance when applied to new data. We will also construct LASSO (least absolute shrinkage and selection operator) regression models for medication adherence and C-MCIs. LASSO regression is a penalized regression method that produces interpretable parsimonious models. These models may provide insight into modifiable factors and tailoring factors that can be the target of future intervention studies.

Predictors for our machine learning models include patient demographics, SDOH, comorbid conditions, medication adherence, and common laboratory tests from the previous year. Some predictors, such as sex and race, have stable values. Other predictors could change substantially over the course of a year. For example, a patient may have several HbA1c measurements in a year, and there may be substantial variance between these measurements. To construct models with good prediction performance, the coding (representation) of predictors must be guided by scientific theory behind the disease mechanism. Our data analyst will work closely with subject matter experts during this process. For example, our tentative choice for representing HbA1c is to include 4 quarterly values in our model, reflecting the fact that each HbA1c measurement represents a weighted average of the blood glucose level in the previous 3 months. For patients missing some HbA1c measurements, we will consider a combination of within-participant (eg, last value carried forward and interpolation) and between-participant (eg, median, mean, and possibly adjusting for covariates) imputation. This process will be iterative and must be conducted carefully.

#### Rigor and Reproducibility

To ensure the reproducibility of data extraction, scripts for the data query will be documented. The high quality of the data set will be the foundation for the rigor of our study. All decisions made regarding data cleaning will be documented and executed via scripts in R statistical software (R Foundation for Statistical Computing), ensuring that the resulting data set for analysis will be of high quality and the entire process will be reproducible. We will use R Markdown in data analysis to ensure that all results are completely reproducible. Our analysis including sex and age as predictors will provide valuable insights into their impact on medication adherence and patient outcomes.

### Aim 3

#### Research Procedures

Key informant interviews will be conducted to learn about the facilitators of and barriers to medication adherence within the context of local SDOH. This important knowledge cannot be discovered through EHR data alone and will provide vital information necessary to develop targeted and tailored interventions designed to improve medication adherence and manage T2D and other cardiometabolic conditions. We will draw a subsample of 90 patients from the larger sample to identify facilitators of and barriers to medication adherence. Our sample will be drawn based on glucose-lowering medication adherence (PDC <0.8 or PDC ≥0.8) and HbA1c control targets (at target, above target, or uncontrolled). To draw our purposeful sample of 90 patients, we will sample 45 patients from each of the two glucose-lowering target medication adherence levels: (1) at target (PDC ≥0.8) and (2) below target (PDC <0.8). Within each of the target medication adherence levels, we will include 15 patients from each of the three HbA1c target control levels: (1) HbA1c ≤7% (at target), (2) HbA1c from >7% to ≤9% (above target), and (3) HbA1c >9% (uncontrolled). These values were selected based on Medicare definitions [40] and American Diabetes Association guidelines [31]. Although we are drawing our sample based only on glucose-lowering medication adherence, we will interview the participants about facilitators and barriers to glucose- and lipid-lowering and antihypertensive medication adherence within the context of SDOH. We are not including all classes of medication (lipid-lowering and antihypertensive) or all C-MCIs (LDL-C and SBP) because it would be too complex. Although 90 patients are a large sample for a theory-generating or phenomenology qualitative study, selected purposefully based on their medication adherence and control, 90 patients responding to our open-ended questions is a reasonable approach. We expect to achieve response saturation for the facilitators of and barriers to medication adherence for the many types of medications used for C-MCIs and with consideration of the individual SDOH that may impact adherence within CNO health care system, which addresses some of the structural SDOH.

#### Design

A qualitative descriptive content analysis design using interviews with open-ended questions will be used to identify facilitators of and barriers to medication adherence within the context of SDOH among CNO patients with T2D.

#### Setting

This study will take place within CNO’s 10.5 county service area to facilitate face-to-face interviews. Qualitative interviews will be conducted by a community health worker from the Choctaw Nation. Interviews will be conducted face-to-face at a convenient location.

#### Sample Eligibility Criteria

We will purposively select a subsample of American Indian patients (documented in CNO EHR) from our larger sample, all of whom have a diagnosis of T2D. The subsample *inclusion criteria* include (1) enrolled tribal member, (2) aged ≥18 years, (3) who have been diagnosed with T2D, (4) live within CNOs 10.5 county service area, (5) who use CNO Tribal Health Services, (6) for whom C-MCI levels and medication adherence level will have been generated from the aim 1 analysis of CNO data, and (7) willing and able to participate in a 60- to 90-minute interview focused on facilitators of and barriers to medication adherence. Although tribal patients who live throughout Oklahoma and the United States use CNO Tribal Health Services, we will *exclude* those residing outside CNO’s 10.5 county service area for this qualitative study. We will exclude tribal members with ESRD, consistent with aim 1 and aim 2 exclusion criteria.

#### Sample Size

We will recruit 90 CNO patients and conduct an open-ended interview per patient. Our sample will include 15 patients from each of the 6 groups, represented by the matrix shown in Figure 2. These interviews will provide a deeper understanding of the facilitators of and barriers to adherence for patients within each of the target medication adherence levels and HbA1c control levels. For example, we will learn from 15 patients who meet the target medication adherence and are at target for HbA1c control their perceptions of what factors make medication adherence difficult and easier. Their perceptions may be different from those within the group with below-target medication adherence and above-target HbA1c control. For these reasons, we divided the sample into 6 different groups. Using guidance from Morse [41], who found that at least 6 participants are needed to understand the essence of an experience, we estimate that 15 interviews per control level will be enough to gain insight and meaning around facilitators and barriers to medication adherence and to reach data saturation [41].

#### Data Collection

We will draw our subsamples as previously described. Once patients are identified, we will provide a coded list of eligible patients from our deidentified data set to CNO information management team. CNO information management team will provide the contact information for 135 individuals directly to CNO community health worker, who will then contact the patients. We expect that from the 135 total available samples in the 6 sampling groups based on medication adherence and C-MCIs, over 80% (108/135) will agree to participate, of which 83.3% (90/108) will follow through to complete the interviews based on our past experience with recruiting for a CNO focus group study [42].

CNO community health worker will contact by telephone the identified eligible patients within each of the sampling groups (labeled 1-6) to introduce the study. Patients who are eligible and interested in participating in the study will be given the option to participate in a face-to-face interview at a convenient site. A structured interview guide using semistructured and open-ended questions with probes will be used to collect in-depth information on facilitators and barriers to glucose- and lipid-lowering and antihypertensive medication adherence. The interview guide will be structured based on each sampling group’s level of medication adherence for all targeted medications as well as levels of C-MCIs.

As part of the interview session, community health workers will ask patients to complete 3 items: demographics, SDOH, and health care use. The patients will be compensated with a gift card of US $100 at the end of the interview. Interviews are expected to last 60 minutes, but owing to the open-ended nature of the probes and to be respectful of different communication styles, such as time to think before answering and telling stories to answer questions, up to an hour and a half may be necessary for adequate discussion. All interviews will be audio-recorded, professionally transcribed verbatim, and validated for accuracy.

#### Measures

The demographic items include questions on age, gender, household income, number of people living at home, housing type, number of bedrooms, employment status, and education level. The SDOH items include questions on transportation, miles to grocery stores and tribal clinics, involvement with CNO cultural activities, use of CNO food distribution, and trust in providers and pharmacists. The medication and health care use history items include questions on medication regimen, length of time on medication, and frequency of provider appointments. Semistructured and open-ended questions will be structured based on medication adherence levels (at target and below target) and HbA1c control (at target, above target, and uncontrolled). Questions include “What is your understanding about the role of medication in managing your T2D?” and “Tell me what makes taking your medication easy or difficult?” Although our interview questions will focus on medication adherence, we will likely learn from patients’ spontaneous comments about their HbA1c, LDL-C, and SBP control through our conversation. This information will be analyzed appropriately.

#### Data Management

Data will be collected electronically, and interviews will be audio-recorded and transcribed. A CNO community health worker will interview patients and collect questionnaire information electronically using data written on a UF research electronic data capture site specifically created for this study. This information will be stored in locked filing cabinets or on computer servers with secure passwords or encrypted electronic storage devices.

All patients will be assigned a code number, and their data will be identified only with the code number. The link of the code numbers to the subject identifiers will be kept separate from the study data. Only CNO study team members will have access to the code or master key. The data will be stored in a controlled-access computer database on a secure UF server. Any hard copy will be stored in a locked office within CNO, scanned, and uploaded to the research electronic data capture site.

#### Data Analysis—Aim 3: Identify Facilitators of and Barriers to Medication Adherence Within the Context of SDOH, EHR-derived Medication Adherence (PDC), and C-MCIs (at Target, Above Target, and for Uncontrolled HbA1c)

Results from the demographic, SDOH, and medication and health care use history questionnaires will be analyzed using descriptive statistics. We will use content analysis, a systematic and objective means of describing a phenomenon, to interpret and analyze the 90 interviews. All interviews will be audio-recorded and professionally transcribed verbatim with accuracy verification. In addition, 10% (9/90) of the transcripts will be rechecked for accuracy. The unit of analysis will be at the word or sentence level. A coding manual will be developed to ensure the consistency of coding [43]. Members of the research team will immerse themselves in the data by reading and rereading interviews. Themes and subthemes of barriers to and facilitators of medication adherence will emerge. Tentative categories will be generated inductively until a final set of categories are developed. Areas of incongruence will be discussed until consensus is reached. We will use a qualitative data management program to analyze and manage the data.

#### Rigor and Reproducibility

We will use rigorous processes including coding checks and audit trails to support reproducibility. Trustworthiness will be addressed through credibility, dependability, confirmability, and transferability [44]. Credibility will be addressed through investigator and data triangulation [44]. Multiple research team members will code, analyze, and interpret the data. Data triangulation will be attained by using different data sets that emerge such as raw data, codes, concepts, and saturation. Dependability and confirmability will be attained through the use of audit trails, which will describe the detailed steps taken throughout the study and notes from team meetings [44,45]. Finally, transferability will be achieved using a codebook with detailed documentation of the coding scheme definitions, coding rules, and examples [44].

We expect to hear rich stories about facilitators of medication adherence such as family support, free medication fills, and importance of medications as well as discussions around barriers to medication adherence such as negative side effects, not believing the medicine will help, and not trusting their provider.

### Timeline

**Figure 3:**
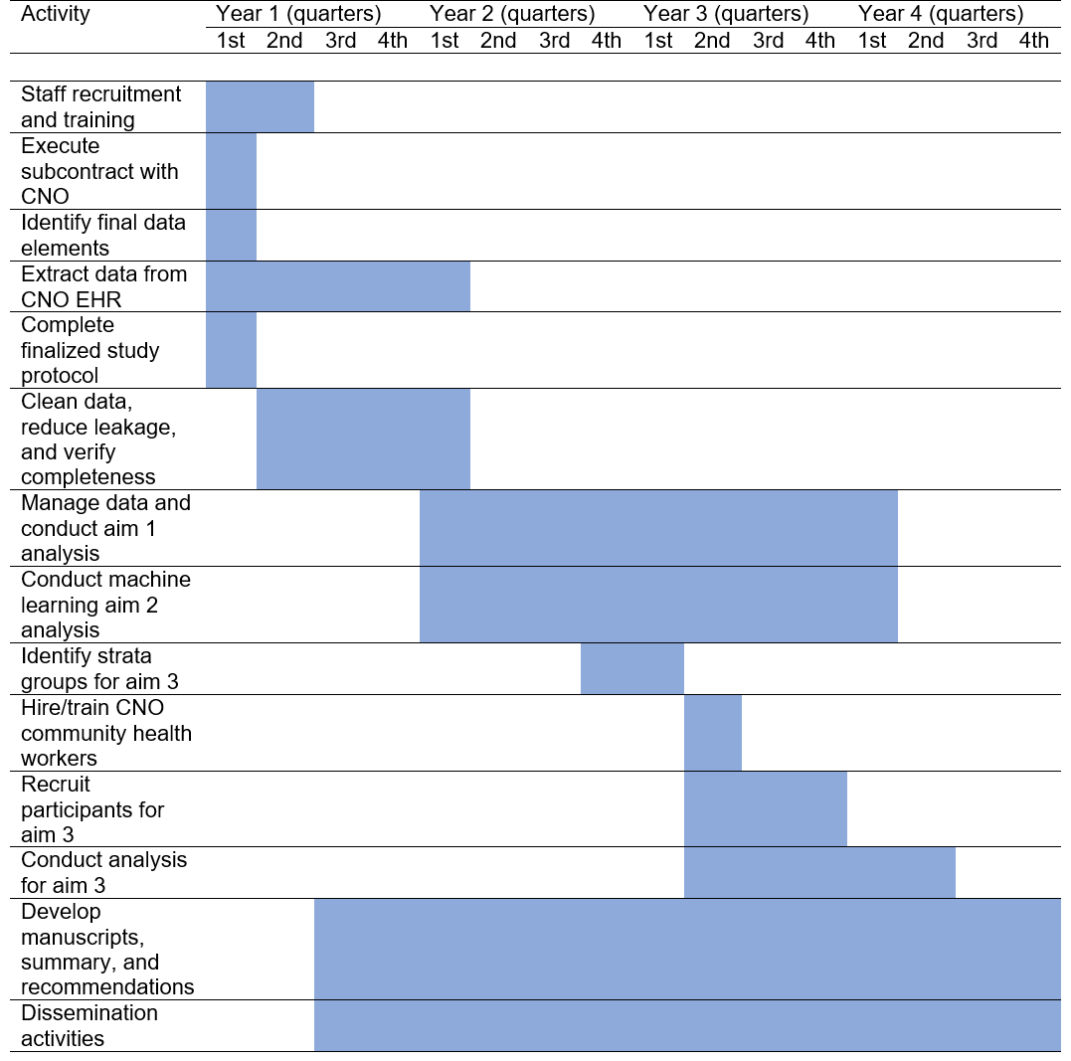
Timeline. CNO: Choctaw Nation of Oklahoma; EHR: electronic health record.

The 4-year timeline for the benchmark for success is presented in Figure 3. We will request and extract CNO data, after the institutional review board (IRB) approval, by month 3. We will complete the study protocol by the end of month 2 and recruitment and training by the end of month 4. We will extract and clean the data, reduce leakage, and verify the data for completeness by the end of the first quarter of year 2. We will complete the analysis of aims 1 and 2 by the end of the first quarter of year 4. On the basis of the data from aim 2, we will identify sampling groups from aim 3 by the first quarter of year 3. We will hire and train CNO community health workers, begin recruitment by the end of the second quarter in year 3, and complete the 90 interviews by the end of year 3 (3-4 per week). We will complete the aim 3 qualitative analysis by the end of the second quarter of year 4. We will finalize manuscripts for all 3 aims and finalize a renewal application by the end of year 4.

### Ethics Approval

The CNO IRB (2,022,001) and UF IRB (02200979) approved the study protocol. We also have an executed data use agreement.

## Results

Funding was obtained in early 2022. The UF and CNO have approved the IRB protocols and executed the data use agreements. Data extraction is in process. We expect to obtain results from aims 1 and 2 in 2024.

## Discussion

### Expected Findings

Our study will provide insights into medication adherence and its potential to predict C-MCIs, which will allow CNO to provide precision behavioral health care to patients by identifying those who may benefit from targeted interventions to prevent future T2D-related complications. High-quality metrics will be available for future CNO program planning and will shape the specific design and sample size of adequately powered future T2D-related studies. Furthermore, understanding the facilitators of and barriers to medication adherence within the context of SDOH will help move us toward eliminating health disparities and reaching health equity for American Indian adults.

### Potential Problems and Alternate Plans

We may experience 2 important issues during the implementation of our study. First, there may be patients diagnosed with T2D who do not have office visits or laboratory results. However, we do not anticipate this being an issue for many patients because CNO health care providers attempt to see patients with elevated HbA1c levels every 3 months and those with well-managed HbA1c levels every 6 months. In addition, patients must visit their primary care provider to receive medication refills. The frequency at which patients fill their medication and follow up with their health care provider during the 4-year period will be informative for intervention development. We focused on American Indian adults aged ≥18 years because of the high incidence and prevalence of T2D in this population across the life span. The prevalence of T2D in youth <18 years is increasing; however, owing to the relatively small number of CNO youth with T2D, we excluded them due to concerns of maintaining anonymity in this study of EHR data.

### Summary

C-MCIs not at target increase the risk for complications in patients with T2D; however, adherence to glucose- and lipid-lowering and antihypertensive drugs can substantially improve C-MCIs and decrease morbidity and mortality. Currently evidence on medication adherence among reservation-dwelling American Indian adults is lacking. Using EHR data from 5970 CNO patients with T2D, we will examine medication adherence and C-MCIs in American Indian adults aged ≥18 years. In addition, we will use a subsample of patients to identify facilitators of and barriers to medication adherence within the context of SDOH. These findings will help us move toward eliminating health disparities and reaching health equity. Findings from this important study will yield insights to improve medication adherence and C-MCIs among American Indian adults with T2D, which is consistent with CNOs’ goal of reducing T2D and its complications (HTN and CVD).

## Appendix

Multimedia Appendix 1 Summary statement.

## Abbreviations

CKD: chronic kidney disease
C-MCI: cardiometabolic control indicator
CNO: Choctaw Nation of Oklahoma
CVD: cardiovascular disease
EHR: electronic health record
ESRD: end-stage renal disease
HbA1c: hemoglobin A1c
HTN: hypertension
ICD-10: International Classification of Diseases, Tenth Revision
IHS: Indian Health Service
IRB: institutional review board
LDL-C: low-density lipoprotein cholesterol
PDC: proportion of days covered
PMN: primary medication nonadherence
PQA: Pharmacy Quality Alliance
SBP: systolic blood pressure
SDOH: social determinants of health
SHS: Strong Heart Study
T2D: type 2 diabetes
UF: University of Florida

## Data Availability

We are using deidentified data under an executed data use agreement with CNO. Under the data use agreement, we do not have the authority to share these data.
