# Supplementary material for: Medication Adherence and Cardiometabolic Control Indicators Among American Indian Adults Receiving Tribal Health Services: Protocol for a Longitudinal Electronic Health Records Study
Source: JMIR Res Protoc. 2022 Oct 24;11(10):e39193. doi: 10.2196/39193 (PMC9641513; doi:10.2196/39193)
Supplement: Multimedia Appendix 1 [file resprot_v11i10e39193_app1.pdf]

SUMMARY STATEMENT

(Privileged Communication)

PROGRAM CONTACT:
Karen Huss
301.594.5970
hussk@mail.nih.gov

Release Date: **10/23/2021**
Revised Date:

Application Number: **1 R01 NR020386-01**

Principal Investigator

SCARTON, LISA

Applicant Organization: UNIVERSITY OF FLORIDA

Review Group: **CDMA**
Clinical Data Management and Analysis Study Section

Meeting Date: **10/14/2021**
Council: **JAN 2022**
Requested Start: **04/01/2022**
RFA/PA: **PA20-185**
PCC: **ACCKH**
Dual IC(s): **MD, DK**

Project Title: **Medication Adherence and Cardio-Metabolic Control Indicators among Adult American Indians Receiving Tribal Health Services**
SRG Action: **Impact Score:31 Percentile:8**
Next Steps: **Visit https://grants.nih.gov/grants/next_steps.htm**
Human Subjects: 30-Human subjects involved - Certified, no SRG concerns
Animal Subjects: 10-No live vertebrate animals involved for competing appl.
Gender: 4A-Gender representation unknown, scientifically acceptable
Minority: 4A-Minority representation unknown, scientifically acceptable
Age: 3A-No children included, scientifically acceptable

Project Year	Direct Costs Requested	Estimated Total Cost
1	346,583	502,760
2	349,679	507,251
3	349,888	507,554
4	348,490	505,526
5	349,967	507,669
TOTAL	1,744,607	2,530,761

ADMINISTRATIVE BUDGET NOTE: The budget shown is the requested budget and has not been adjusted to reflect any recommendations made by reviewers. If an award is planned, the costs will be calculated by Institute grants management staff based on the recommendations outlined below in the COMMITTEE BUDGET RECOMMENDATIONS section.
EARLY STAGE INVESTIGATOR
NEW INVESTIGATOR

1R01NR020386-01 Scarton, Lisa

COMMITTEE BUDGET RECOMMENDATIONS
EARLY STAGE INVESTIGATOR
NEW INVESTIGATOR

RESUME AND SUMMARY OF DISCUSSION: In this application, the investigators propose to investigate the relationship between medication adherence and cardio-metabolic control indicators (C-MCI) using Choctaw Nation of Oklahoma (CNO) population electronic health records data (EHR) by developing models that predict future C-MCI and identify facilitators to and barriers of medication adherence. The review committee considered the scientific foundation of the study well supported by their strong preliminary data and also by scientific literature. They thought the study to be significant as there is limited evidence of longitudinal medication adherence or cardiometabolic control in American Indian populations with diabetes residing on reservations, and the proposed study if successful, would help understand the current problems faced by CNO populations with diabetes. They considered the investigative team well experienced with complementary strengths and prior collaborations, while limited experience in dealing with EHR diabetes data, and overlapping expertise amongst the investigative team. Other noted strengths include the use of EHR data to examine medication adherence and C-MCI in a large community setting to be innovative, use of machine learning approaches for prediction in a population where no existing prediction models exists, well defined cohort, use of appropriate sample size and power calculations, and well described methods towards developing predictive models. However, concerns were noted in the rigor of the approach. These include the generalizability of the study beyond the CNO population, limited details on the forecasting model and the system level integration, inadequate details on the external validation datasets, limited details on the social determinants of health variables in the modeling, and minimal information on the issues with analyzing medication adherence over longitudinal data. Following discussion, the review committee agreed the study would have a moderately high impact to improve medication adherence and C-MCI among American Indian population.

DESCRIPTION (provided by applicant): American Indians (AIs) have the highest prevalence of type 2 diabetes (T2D) of any racial or ethnic group and experience high rates of co-morbidities such as obesity, cardiovascular disease (CVD), and chronic kidney disease (CKD). Uncontrolled cardio-metabolic risk factors--insulin resistance resulting in impaired glucose tolerance, dyslipidemia, and hypertension (HTN)--increase mortality risk. Mortality is significantly reduced by glucose- and lipid-lowering, and antihypertensive medication adherence. Medication adherence is low among AIs living in non-Indian Health Services (IHS) healthcare settings. Virtually nothing is known about the nature and extent of medication adherence among reservation-dwelling AIs who primarily receive their medications without cost from IHS/tribal facilities. Electronic health records (EHR) offer a rich but underutilized data source about medication adherence and its potential to predict Cardio-Metabolic Control Indicators (C-MCI) such as HbA1c, LDL-C (Low Density Lipoprotein), SBP (Systolic Blood Pressure). With the support of Choctaw Nation of Oklahoma (CNO), we will address this oversight by using EHR data generated by this large, state-of- the-art tribal healthcare system to investigate C-MCI. The objective of our R01 application is to characterize the relationships among medication adherence (antihypertensive, glucose- and lipid-lowering drugs) and C- MCI (HbA1c ≤7%, LDL-C <100 mg/dL, and SBP <130 mm Hg), patient demographics (e.g., age, sex, SDOH, residence location) and co-morbidities (e.g., CVD, BMI>30, CKD) as well as the relationship of each C-MCI with patient demographics and co-morbidities from the tribe's EHR (2018-2021) for the 5,970 CNO patients who have T2D. Employing machine learning techniques, we will develop models to predict future (2019-2021) C-MCI based on the previous year medication adherence, patient demographics, co- morbidities, and common labs (e.g., lipid panel). Lastly, key informant interviews will explore facilitators of and barriers to medication adherence within

the context of local social determinants of health (SDOH) that are not available in the EHR. Our specific aims are to: (1) Determine the bivariate relationships between (a) medication adherence and C-MCIs, demographics, and co-morbidities; (b) each C-MCI and demographics and co-morbidities; (2) Develop machine-learning models (e.g., random forest, nearest neighbors, others) for predicting future (2019-2021) C-MCI from the previous year medication adherence, demographics, co- morbidities, and common labs; and (3) Identify facilitators of and barriers to medication adherence within the context of SDOH, EHR-derived medication adherence (PDC) and C-MCI (at target, above target, and for HbA1c uncontrolled). We will share our findings with CNO leaders and other stakeholders, who will guide the translation of the results into recommendations for evaluating T2D management and complication prevention programs. Our findings will yield insights to improve medication adherence and C-MCI among AIs, consistent with CNO's State of the Nation's Health Report 2017 goal of reducing T2D and its complications.

PUBLIC HEALTH RELEVANCE: Our long-term goal is to reduce type two diabetes (T2D)-related health disparities within Choctaw Nation of Oklahoma (CNO) communities and prevent future complications such as cardiovascular disease and hypertension. Toward that goal, we propose to analyze four years of electronic medical record data generated by a large, state-of-the-art tribal healthcare system to investigate the relationship between medication adherence and cardio-metabolic control indicators (C-MCI), to develop models that predict future C-MCI, and identify facilitators to and barriers of medication adherence. Our results will yield insight to improve medication adherence and C-MCI among American Indians, consistent with CNO's State of the Nation's Health Report 2017 goal of reducing T2D and its complications and to improve C-MCI among CNO patients and other American Indian communities based on future research with data from additional tribes.

CRITIQUE 1

Significance: 4
Investigator(s): 3
Innovation: 3
Approach: 3
Environment: 1

Overall Impact: This project, titled "Medication Adherence and Cardio-Metabolic Control Indicators among Adult American Indians Receiving Tribal Health Services", is a 5-year initial submission of an R01 by an early stage new investigator from the University of Florida, with a team of University of Florida researchers, alongside co-I from the Choctaw Nation, and Western Carolina University. Broadly, the project aims to obtain EHR records of individuals with T2D from a large tribal healthcare system, extracting data related to (1) medication adherence to glucose- and lipid-lowering, and antihypertensive drugs; (2) Cardio-Metabolic Control Indicators (C-MCI)--e.g., HbA1c ≤7%, low density lipoprotein LCL-C<100 mg/dL and systolic blood pressure<130mmHg; (3) patient demographics (e.g., age, sex, SDOH, residence location), and (4) co-morbidities (e.g., BMI>30, cardiovascular disease CVD, chronic kidney disease CKD). These data are modelled as cross-sections to discover associative relationships in Aim 1, and then in Aim 2 the data is taken longitudinally to build forecasting models that predict changes in CMC-I. Informant interviews are engaged in Aim 3 to collect and analyze qualitative contextual data on facilitators of and barriers to medication adherence within the context of local SDOH. There are many strong aspects of the proposal, including solid preliminary results in support of the scientific premise, particularly Aims 1 and 3. Sufficient evidence is presented showing that adherence to antihypertensive, glucose- and lipid-lowering medications can substantially improve C-MCI, and this can, in turn, reduce

the risk of complications like CVD and CKD in AIs with T2D. Preliminary findings by the PI have quantified variability of HbA1c control levels and the variability of antihypertensive, glucose- and lipid-lowering medication adherence in AI patients with T2D; these provide a good foundation for this project. The project is innovative in that it addresses a data gap. As it stands, there is very little longitudinal data on adherence or C-MCI for AIs with T2D living on tribal lands and receiving care from a tribal healthcare system, nor is there much data on association between SDOH and medication adherence for AIs. The proposal is supported by a stellar multidisciplinary research team which has prior collaborations on these topics, including with the Choctaw Nation. At the same time, a few moderate concerns arose which dampened my enthusiasm. With regards to significance, the proposal did not make a case that conclusions drawn from the study would be generalizable beyond the Chocktaw Nation (or even how generalizability would be assessed). Of the 3 Aims, the second Aim which seeks to forecast CMC-I was found to be the weakest and hardest to evaluate in all regards mainly due to under-specification. The premise was unclear, but more crucially specificity and details of research methodology were lacking, as was a clear linkage between Aim 2 outputs and the interventions that a forecasting model would purportedly facilitate remain underspecified. There also appeared to be some overlap in team members' expertise, without a clear division of roles or coordination plan where overlap might be present. These moderate concerns diminished my enthusiasm somewhat, for an otherwise well written and important project.

1. Significance:

Strengths

- If successful, the project will move us toward a better understanding of health disparities and will create new knowledge that may inform policies to improve medication adherence and C-MCI among AIs with T2D.
- The scientific premise of the project is solid, resting on the fact that (1) Adherence to antihypertensive, glucose- and lipid-lowering medications can substantially improve C-MCI, and (2) in turn, this can reduce the risk of complications like CVD and CKD in AIs with T2D. These claims are adequately shown by reference to existing prior research.
- Preliminary findings by the project team provide a strong foundation for this project. Earlier pilot has quantified variability of HbA1c control levels and the variability of antihypertensive, glucose- and lipid-lowering medication adherence in AI patients with T2D.

Weaknesses

- Insufficient arguments for the generalizability of conclusions to AI beyond the Choctaw Nation. To what extent are the CNO typical, and what is the strategy for validation of conclusions to the larger at-risk populations of AI?
- Aim 2 impacts appear inflated. Translational linkages between a forecasting model and actual data driven individual-level behavioral or system-level intervention strategies remain unclear. Suppose that poor adherence in 2017 is found to be the strongest predictor in an ML model of worsening CMC-I in later years. How is that useful or interesting or actionable?

2. Investigator(s):

Strengths

- PI is an enrolled tribal member and has worked with tribal leaders for more than 5 years
- Project includes 2 pharmacists who have experience with CNO, and also expertise in medication adherence among individuals with T2D

- Project includes lead for IHS Special Diabetes Program for Indians
- Team includes 2 experienced ML EHR researchers and a statistician

Weaknesses

- The team is not small, and there appears to be insufficiently justified overlapping expertise across team members in several areas (modeling, pharmacy, etc). The roles of each member, as well as coordination in overlap areas, are not clearly described in the research strategy or biosketches.

3. Innovation:

Strengths

- Electronic health record (EHR) systems at these facilities offer a rich but underutilized data source about medication adherence and its potential to predict Cardio-Metabolic Control Indicators (C-MCI); e.g., HbA1c, LDL-C (low density lipoprotein), SBP (systolic blood pressure)
- There is very little existing longitudinal data on adherence or C-MCI for AIs with T2D living on tribal lands and receiving care from a tribal healthcare system
- Little existing data on association between SDOH and medication adherence for AIs

Weaknesses

- The techniques for mathematical modeling are fairly routine.
- There is insufficient support for (or a definition of) success associated with Aim 2.

4. Approach:

Strengths

- Scientific rigor is adequate. The Choctaw Nation EHR reports ~6K patients with T2D in 2017. The project will analyze the EHR data of these patients in 2017 subsequent years (2018-2021) and apply machine learning to develop prediction models for future C-MCIs based on medication adherence, patient demographics, comorbidities, and common labs (e.g., lipid panel) from the previous year. The EHR data will also be mined to discover relationships between (1) medication adherence (to glucose- and lipid-lowering, and antihypertensive drugs) and (2) Cardio-Metabolic Control Indicators (C-MCI)--e.g., HbA1c ≤7%, low density lipoprotein LCL-C<100 mg/dL and systolic blood pressure<130mmHg and (3) patient demographics (e.g., age, sex, SDOH, residence location) and (4) comorbidities (e.g., BMI>30, CVD, CKD).
- Clear exposition as to how adherence is assessed: using prescription and pharmacy refill data to calculate the proportion of days covered with medications.
- Informant interviews are engaged in Aim 3 to collect qualitative contextual data on facilitators of and barriers to medication adherence within the context of local SDOH. Qualitative methods are clearly described.
- Inclusion of both at-target (resp. below-target) sub-populations to discover facilitators (resp. barriers) to adherence is commendable.
- Three Aims are largely decoupled, lowering risk of project failure.
- Sex is adequately considered in the analyses.

Weaknesses

- Aim 2 execution is missing sufficient details concerning hyper-parameters, feature selection, and scaling.
- ML regressors output in Aim 2 will be black box and likely uninterpretable. Unclear how these forecasts would be incorporated into an intervention on the ground.
- The proposal timeline asserts “Based on data from aim 2 we will identify sampling groups from aim 3 by the second quarter of year 3”. It is unclear how or why a predictive model should help in the sample selection for Aim 3. All that is needed is to cluster the ~6K and take representatives from each cluster. As such, it is unclear why Aim 3 analyses must wait for Aim 2 to be completed.

5. Environment:

Strengths

- The environment is excellent and sufficient to achieving project success.

Weaknesses

- None noted.

Protections for Human Subjects:

Acceptable Risks and/or Adequate Protections

- Aims 1 and 2 use de-identified EHR data. Aim 3 draws 90 patients to identify facilitators of and barriers to medication adherence. The CNO CHW mediates between the study team and the participants during recruitment. Data is descriptive with open-ended questions to identify facilitators of and barriers to medication adherence within the context of SDOH among AI patients with T2D.

Inclusion Plans:

- Sex/Gender: Distribution justified scientifically
- Race/Ethnicity: Distribution justified scientifically
- For NIH-Defined Phase III trials, Plans for valid design and analysis: Not applicable
- Inclusion/Exclusion Based on Age: Distribution justified scientifically
- Excluding children < 18

Vertebrate Animals:

Not Applicable (No Vertebrate Animals)

Biohazards:

Not Applicable (No Biohazards)

Resource Sharing Plans:

Acceptable

- Acceptable, given this is data owned by the Choctaw Nation

Budget and Period of Support:

Budget Modifications Recommended (in amount/time)

Recommended budget modifications or possible overlap identified:

- Three years to clean and analyze EHR records for Aims 1 and 2 is excessive.
- Aim 3 analyses do not need to wait for Aim 2 to complete.
- The project duration can be reduced by at least 1 year, if not more.

CRITIQUE 2

Significance: 2
Investigator(s): 3
Innovation: 1
Approach: 4
Environment: 1

Overall Impact: This proposal focuses on characterizing the relationships among medication adherence and cardiometabolic control (a1c, ldl, sbp), developing a predictive model for future control, and qualitative interviews among the Choctaw Nation of Oklahoma's population with type 2 diabetes. The major score-driving factors with the significance, innovation, investigators, and approach. The significance was considered high because of the scant data on longitudinal medication adherence and cardiometabolic control in this population. The innovation was considered high because the potential information gathered from Aims 1 and 3 would likely greatly advance our understanding of medication adherence for this population. The investigative team was considered high-moderate; overall the team, and PI (ESI) brought a number of complementary strengths. However, the lack of a team member with clear experience doing longitudinal EHR studies in diabetes could lead to challenges completing this Aim in a timely manner. The approach was judged to be moderate, and while many aspects were good (the longitudinal EHR data with dispensing information, Aim 3 being well-described), there was a lack of detailed explanation for decisions in the analysis of Aim 1 (lack of mediator, effect modification, emphasis on bivariate analysis), lack of information on what SDOH variables were available in the EHR, lack of detailed explanation for how some of the complexity of longitudinal EHR medication adherence data (dose adjustments, mail order, dispensing vs. taking) would be managed. Overall, because of these factors, the impact was considered to be high, with a few reservations.

1. Significance:

Strengths

- This research question is important because there is scant evidence of longitudinal medication adherence or cardiometabolic control in AIAN populations with diabetes residing on reservations
- Similarly, examination of patient facilitators/barriers for diabetes medication adherence in relationship to a1c control has not been previously done at this scope for this population.
- Prior research provides strong evidence that there is a need to study this issue further.
- Assuming that this project is successful, the output from this proposal, especially Aim 1 and 3, could lead to important advances in our understanding of the current problems faced for CNO populations with diabetes.

Weaknesses

- Aim 2 focuses on developing a machine learning model for predicting future cardio metabolic control indicators based on the previous year's patient characteristic, and while this will be new, the lack of predicting future uncontrolled populations seems to be a second order problem, when large populations are currently poorly controlled.

2. Investigator(s):

Strengths

- Dr. Scarton is an early stage nurse investigator with the appropriate experience and training to complete the proposal. She is a tribal citizen of CNO, which will help for establishing trust and has a history of doing research with CNO for >5 years. She has previous qualitative research experience partnering with this community. She also has studied T2D management and prevention interventions, which is relevant for this work.
- Dr. Circles provides complementary expertise as site PI for the Choctaw Nation and is a pharmacist and has done research medication reconciliation and adherence in diabetes, which is relevant expertise.
- Dr. Troy is a practicing endocrinologist, which will be helpful for the clinical context and has experience in T2D health services research.
- Dr. Goins is an epidemiologist and has extensive mentorship experience. She also has expertise in AIAN research, which will help to provide cultural context.
- Dr. Manson is a leader in the field of diabetes research in this population and insights will be helpful.
- Dr. Yao has the experience necessary to do the statistical analyses necessary for Aims 1 and 2.
- Dr. Segal also provides expertise on medication adherence and diabetes in vulnerable groups.

Weaknesses

- Dr. Wilkie's unique expertise and contribution for this proposal is less clear. While being an accomplished nurse researcher, her content and research experience is in pain, cancer, tablet technology, and disparities. She does mention expertise in EHR data which will be important, but the extent of the experience is unclear.
- Longitudinal EHR data is quite complicated, and it is not clear that the investigative team has experience with this issue and for diabetes or cardiometabolic disease, which is different than sickle cell disease or cancer.

2. Innovation:

Strengths

- This proposal would provide a foundational understanding of longitudinal medication adherence and their associations with cardiometabolic risk factor control in a large community of reservation dwelling AIs with diabetes. This type of information is needed to design interventions to improve health outcomes for this population who suffers from major healthcare disparities.
- Detailed qualitative examination of medication adherence and levels of A1C control in this population is a major innovation which could lead to a shift in research interventions in the future.

Weaknesses

- None noted by reviewer.

3. Approach:

Strengths

- The approach to Aim 2 is appropriate for the goal of developing a predictive model.
- The EHR data will have data on dispensing data which are often hard to gather
- The 4 year dataset will provide a long enough time to do a good analysis for Aim 1 and 2.
- Potential problems are addressed.
- A thorough an appropriate discussion of the methods for Aim 3 provide good evidence of the scientific rigor of this aim.

Weaknesses

- In Aim 1, there is an emphasis on a bivariate analysis, but it is not clear why only a bivariate analysis is being used, which affects the scientific rigor of Aim 1. There doesn't not seem to be a discussion of evaluating for mediators, effect modifiers, or interaction terms, which will all be issues for these kinds of data.
- There is an emphasis on target of control in the Aim1 and 2 analyses, but these values are continuous and there is an understanding in the ADA guidelines that individualized glycemic control, and for other targets are important. The scientific rigor would be strengthened if they considered that the targets of control are binary cutoffs of a continuous variable in their approach.
- It is unclear what SDOH variables will be included in the modeling of Aims 1 and 2, especially because typically few SDOH are included in the EHR.
- Medication adherence is well-described, but some of the more complex issues with analyzing this issue over longitudinal data are not thoroughly described – for example, the use of mail-order, which affects adherence and can lead to discrepancies between medication taking vs. medication dispensed; how drug switching would be considered, and why if patients are supposed to be on 2 or 3 medications for a condition why adherence to only 1 medication would be considered sufficient.
- In Aim 3, there appears to be a pre-supposed 15 participants per subgroup, of which there are 6 subgroups. However, as the proposal acknowledges, fewer than 15 participants may be all that is necessary to achieve theme saturation. A minor suggestion would be to consider theme saturation achievable within each of the 6 groups at whatever N is necessary, in accordance with the accepted methodology for this approach.
- Sex as a biological variable is not discussed.

5. Environment:

Strengths

- The environment is well-described and the PI and Co-Is have the physical space and computing resources necessary to complete the project.

Weaknesses

- None noted by reviewer.

Protections for Human Subjects:
Acceptable Risks and/or Adequate Protections

Inclusion Plans:
- Sex/Gender: Distribution justified scientifically
- Race/Ethnicity: Distribution justified scientifically
- For NIH-Defined Phase III trials, Plans for valid design and analysis: Not applicable
- Inclusion/Exclusion Based on Age: None noted

Vertebrate Animals:
Not Applicable (No Vertebrate Animals)

Biohazards:
Not Applicable (No Biohazards)

Budget and Period of Support:
Recommend as Requested

CRITIQUE 3

Significance: 2
Investigator(s): 3
Innovation: 2
Approach: 4
Environment: 2

Overall Impact: American Indians have among the highest rates of cardiometabolic disorders among Americans, however, little is known opportunities to improve their health through medication adherence and other comorbid and clinical/demographic factors. The goal of this proposal is to: (1) characterize the relationship between relevant health factors, (2) develop prediction models; and (3) identify barriers and facilitators of cardiometabolic health compliance in the context of SDoH. The rigor of the prior research is moderately strong although relatively scant, which is representative of the dearth of high quality data in this population. The investigator team includes a CNO member and strong support from senior investigators with experience related to AI-relevant health outcomes and disparities. The approach is feasible and well-described. I believe the significance of this research is high. Moderate weaknesses related to the lack of external validation, incomplete descriptions of the SDoH identifiable within EHR data, and the lack of well-established prior collaboration are addressable.

1. Significance:

Strengths

- American Indians (AI) experience the highest prevalence of DM in the US. Medication adherence, a key controlling measure for cardiometabolic disease, is also low in AI communities.
- The rigor of prior research is strong; however, only small or less generalizable studies are available that identify key factors for medication adherence and cardiometabolic risk factors for disease. Prior data show that access, travel, and medication side effects concerns were relevant to med adherence in CNO.
- EHR data from AI communities allows for the evaluation of medication adherence, alongside demographics, comorbidities, and laboratory data. Almost nothing is known about medication adherence, a key mediator of disease control, in AI communities.

Weaknesses

- Lack of external validation dataset may limit generalizability beyond CNO.

2. Investigator(s):

Strengths

- PI is an early stage investigator but shows an established track record of working with CNO on cardiometabolic disease. The PI is a tribal member of CNO which significantly improves the relevance and impact of the study's findings. PI does have track record in DM2 research in the American Indian population.
- Investigator team includes pharmacist from CNO and experts in American Indian healthcare, DM outcomes studies using EHR data, health equity, biostatistics

Weaknesses

- Investigators do not demonstrate a strong history of collaboration with the PI which may present a risk to the research given its span across multiple institutions.

3. Innovation:

Strengths

- This will be one of the first studies using EHR data to examine medication adherence in AI populations, including younger adults.
- ML approaches for prediction, while not novel in and of themselves, will be applied to these data in a new population in which there are no existing prediction models.
- Study will involve direct contact with CNO members to provide qualitative feedback.

Weaknesses

- Application would be strengthened by having more than one AI community represented.

4. Approach:

Strengths

- Clearly defined cohort and well described sample size/power calculations based on 2017 data from CNO EHR.

- Clear and feasible data to be extracted from EHR. PDC measure used is standard for industry.
- Aim 2 ML methods are industry standard and well described.

Weaknesses

- Uncertain expected frequency of missing lab value data which are accurately defining the outcomes measures of C-MCI.
- Aim 2 prediction will use SDoH data as predictor, however, these are not well described nor are they available in Table 3. The SDoH identified in Aim 3 would not be available in EHR data.
- Proposal would be tremendously strengthened by use of an external validation dataset for models developed in Aim 2.
- Aim 3 approach to key informant invitation may systematically exclude patients who have poor access to care (SDoH) and who are not having their DM2 actively treated or tested for.

5. Environment:

Strengths

- This proposal brings together the University of Florida, the Choctaw Nation of Oklahoma, and Western Carolina University.
- Unique access to detailed data from EHR in AI community of CNO. Support letter from CNO executive officer confirming continuation of existing research collaboration.
- Strong support from leading investigators in AI health.

Weaknesses

- None noted by reviewer.

Protections for Human Subjects:

Acceptable Risks and/or Adequate Protections

- Appropriate

Inclusion Plans:

- Sex/Gender: Distribution justified scientifically
- Race/Ethnicity: Distribution justified scientifically
- For NIH-Defined Phase III trials, Plans for valid design and analysis: Not applicable
- Inclusion/Exclusion Based on Age: Distribution justified scientifically
- From age 18 and above given low incidence of DM type 2 in AI population below 18. Study exclusively conducted among AI patients.

Vertebrate Animals:

Not Applicable (No Vertebrate Animals)

Biohazards:

Not Applicable (No Biohazards)

Resource Sharing Plans:

Acceptable

Budget and Period of Support:

Recommend as Requested

THE FOLLOWING SECTIONS WERE PREPARED BY THE SCIENTIFIC REVIEW OFFICER TO SUMMARIZE THE OUTCOME OF DISCUSSIONS OF THE REVIEW COMMITTEE, OR REVIEWERS' WRITTEN CRITIQUES, ON THE FOLLOWING ISSUES:

PROTECTION OF HUMAN SUBJECTS: ACCEPTABLE

INCLUSION OF WOMEN PLAN: ACCEPTABLE

INCLUSION OF MINORITIES PLAN: ACCEPTABLE

INCLUSION ACROSS THE LIFESPAN: ACCEPTABLE

COMMITTEE BUDGET RECOMMENDATIONS:
Reviewers noted that three years to clean and analyze EHR records for Aims 1 and 2 is excessive. Also, the Aim 3 analyses do not need to wait for Aim 2 to complete. The project duration can be reduced by at least 1 year, if not more.

Footnotes for 1 R01 NR020386-01; PI Name: Scarton, Lisa

NIH has modified its policy regarding the receipt of resubmissions (amended applications).See Guide Notice NOT-OD-18-197 at https://grants.nih.gov/grants/guide/notice-files/NOT-OD-18-197.html. The impact/priority score is calculated after discussion of an application by averaging the overall scores (1-9) given by all voting reviewers on the committee and multiplying by 10. The criterion scores are submitted prior to the meeting by the individual reviewers assigned to an application, and are not discussed specifically at the review meeting or calculated into the overall impact score. Some applications also receive a percentile ranking. For details on the review process, see http://grants.nih.gov/grants/peer_review_process.htm#scoring.

MEETING ROSTER

Clinical Data Management and Analysis Study Section
Healthcare Delivery and Methodologies Integrated Review Group
CENTER FOR SCIENTIFIC REVIEW
CDMA
10/14/2021 - 10/15/2021

Notice of NIH Policy to All Applicants: Meeting rosters are provided for information purposes only. Applicant investigators and institutional officials must not communicate directly with study section members about an application before or after the review. Failure to observe this policy will create a serious breach of integrity in the peer review process, and may lead to actions outlined in NOT-OD-14-073 at https://grants.nih.gov/grants/guide/notice-files/NOT-OD-14-073.html, NOT-OD-15-106 at https://grants.nih.gov/grants/guide/notice-files/NOT-OD-15-106.html, and NOT-OD-18-115 at https://grants.nih.gov/grants/guide/notice-files/NOT-OD-18-115.html, including removal of the application from immediate review.

CHAIRPERSON(S)

PAYNE, PHILIP R O, PHD
PROFESSOR AND DIRECTOR
INSTITUTE FOR INFORMATICS
SCHOOL OF MEDICINE
WASHINGTON UNIVERSITY
ST LOUIS, MO 63110

MEMBERS

ADHIKARI, SAMRACHANA, PHD *
DEPARTMENT OF POPULATION HEALTH
DIVISION OF BIOSTATISTICS
NYU SCHOOL OF MEDICINE
NEW YORK CITY, NY 10016

BAGCI, ULAS, PHD *
ASSOCIATE PROFESSOR
RADIOLOGY AND BIOMEDICAL ENGINEERING DEPARTMENT
FEINBERG SCHOOL OF MEDICINE
NORTHWESTERN UNIVERSITY
CHICAGO, IL 60611

BECKETT, LAUREL A, PHD
DISTINGUISHED PROFESSOR EMERITA
DEPARTMENT OF PUBLIC HEALTH SCIENCES
SCHOOL OF MEDICINE
UNIVERSITY OF CALIFORNIA, DAVIS
DAVIS, CA 95616

BIAN, JIANG, PHD
ASSOCIATE PROFESSOR
DEPARTMENT OF HEALTH OUTCOMES
AND BIOMEDICAL INFORMATICS
COLLEGE OF MEDICINE
UNIVERSITY OF FLORIDA
GAINESVILLE, FL 32610

CANAHUATE, GUADALUPE, PHD
ASSOCIATE PROFESSOR
DEPARTMENT OF ELECTRICAL AND COMPUTER ENGINEERING
UNIVERSITY OF IOWA
IOWA CITY, IA 52242

CANNESSON, MAXIME, PHD
PROFESSOR AND CHAIR
DEPARTMENT OF ANESTHESIOLOGY
AND PERIOPERATIVE MEDICINE
UNIVERSITY OF CALIFORNIA, LOS ANGELES
LOS ANGELES, CA 90095

CARLSON, NICHOLE, PHD *
PROFESSOR
DEPARTMENT OF BIOSTATISTICS
AND INFORMATICS
UNIVERSITY OF COLORADO
DENVER, CO 80203

CATO, KENRICK DWAIN, PHD
ASSISTANT PROFESSOR
DEPARTMENT OF EMERGENCY MEDICINE
COLUMBIA UNIVERSITY
NEW YORK, NY 10033

DIAZ GARELLI, FRANCK, PHD *
ASSISTANT PROFESSOR
DEPARTMENT OF PUBLIC HEALTH SCIENCES
UNIVERSITY OF NORTH CAROLINA AT CHARLOTTE
CHARLOTTE, NC 28223

DRAWZ, PAUL ENGLUND, MD *
ASSOCIATE PROFESSOR
DEPARTMENT OF MEDICINE
DIVISION OF RENAL DISEASES AND HYPERTENSION
UNIVERSITY OF MINNESOTA
MINNEAPOLIS, MN 55414

DUNLOP, BOADIE W, MD
PROFESSOR
DEPARTMENT OF PSYCHIATRY AND BEHAVIORAL SCIENCES
SCHOOL OF MEDICINE
EMORY UNIVERSITY
ATLANTA, GA 30329

FRANCESCHINI, NORA, MD, MPH *
PROFESSOR
DEPARTMENT OF EPIDEMIOLOGY
UNIVERSITY OF NORTH CAROLINA AT CHAPEL HILL
CHAPEL HILL, NC 27514

GRANDO, MARIA ADELA, PHD *
ASSOCIATE PROFESSOR
BIOMEDICAL INFORMATICS, COLLEGE OF HEALTH SOLUTION
COLLEGE OF MEDICINE MAYO CLINIC
ARIZONA STATE UNIVERSITY
PHOENIX, AZ 85008

HASSANPOUR, SAEED, PHD
ASSOCIATE PROFESSOR OF BIOMEDICAL DATA SCIENCE AND EPIDEMIOLOGY
DEPARTMENT OF BIOMEDICAL DATA SCIENCE AND EPIDEMIOLOGY, GEISEL SCHOOL OF MEDICINE
DARTMOUTH COLLEGE
LEBANON, NH 03756

HERNANDEZ-BOUSSARD, TINA, PHD
ASSOCIATE PROFESSOR OF MEDICINE
BIOMEDICAL DATA SCIENCE AND SURGERY
SCHOOL OF MEDICINE
STANFORD UNIVERSITY
STANFORD, CA 94305

HIMES, BLANCA E, PHD
ASSOCIATE PROFESSOR
DEPARTMENT OF BIOSTATISTICS, EPIDEMIOLOGY AND INFORMATICS
PERELMAN SCHOOL OF MEDICINE
UNIVERSITY OF PENNSYLVANIA
PHILADELPHIA, PA 19104

HOCHHEISER, HARRY S, PHD *
ASSOCIATE PROFESSOR
DEPARTMENT OF BIOMEDICAL INFORMATICS
INTELLIGENT SYSTEMS PROGRAM
SCHOOL OF COMPUTATION AND INFORMATION
UNIVERSITY OF PITTSBURGH
PITTSBURGH, PA 15206

HORVITZ-LENNON, MARCELA V, MD, MPH
PROFESSOR, SENIOR PHYSICIAN SCIENTIST
RAND CORPORATION
BOSTON, MA 02116

KENNEDY, RICHARD E, MD, PHD
ASSOCIATE PROFESSOR
DEPARTMENT OF MEDICINE
UNIVERSITY OF ALABAMA
BIRMINGHAM, AL 35294

KHAN, BILAL, PHD
HAPPOLD PROFESSOR OF SOCIOLOGY AND COMPUTER SCIENCE
DEPARTMENTS OF SOCIOLOGY AND COMPUTER SCIENCE
UNIVERSITY OF NEBRASKA-LINCOLN
LINCOLN, NE 68588

KOLACHALAMA, VIJAYA B, PHD *
ASSISTANT PROFESSOR
DEPARTMENT OF MEDICINE
COMPUTATIONAL BIOMEDICINE
BOSTON UNIVERSITY
BOSTON, MA 02118

LAITEERAPONG, NEDA, MD
ASSOCIATE PROFESSOR
DEPARTMENTS OF MEDICINE AND PSYCHIATRY AND BEHAVIORAL NEUROSCIENCE
UNIVERSITY OF CHICAGO
CHICAGO, IL 60637

LI, LANG, PHD
PROFESSOR AND CHAIR
DEPARTMENT OF BIOMEDICAL INFORMATICS
COLLEGE OF MEDICINE
THE OHIO STATE UNIVERSITY
COLUMBUS, OH 43210

LIU, FEIFAN, PHD *
ASSISTANT PROFESSOR
DEPARTMENT OF POPULATION AND QUANTITATIVE HEALTH SCIENCES
DEPARTMENT OF RADIOLOGY
UNIVERSITY OF MASSACHUSETTS MEDICAL SCHOOL
WORCESTER, MA 01605

LIU, VINCENT, MD *
RESEARCH SCIENTIST AND REGIONAL DIRECTOR
HOSPITAL ADVANCED ANALYTICS
DIVISION OF RESEARCH
KAISER FOUNDATION RESEARCH INSTITUTE
OAKLAND, CA 94612

LOH, JI MENG, PHD *
ASSOCIATE PROFESSOR
DEPARTMENT OF MATHEMATICAL SCIENCES
NEW JERSEY INSTITUTE OF TECHNOLOGY
NEWARK, NJ 07102

MCMILLAN, ALAN BLAIR, PHD *
ASSOCIATE PROFESSOR
DEPARTMENTS OF RADIOLOGY
UNIVERSITY OF WISCONSIN–MADISON
MADISON, WI 53706

MYNENI, SAHITI, PHD *
ASSOCIATE PROFESSOR
SCHOOL OF BIOMEDICAL INFORMATICS
UNIVERSITY OF TEXAS
HEALTH SCIENCES CENTER
HOUSTON, TX 77030

OBEID, JIHAD, MD *
PROFESSOR
PUBLIC HEALTH SCIENCES
UNIVERSITY OF SOUTH CAROLINA
CHARLESTON, SC 21763

PEDOIA, VALENTINA, PHD *
ASSISTANT PROFESSOR
DEPARTMENT OF RADIOLOGY AND BIOMEDICAL IMAGING
UNIVERSITY OF CALIFORNIA, SAN FRANCISCO
SAN FRANCISCO, CA 94158

POPESCU, MIHAIL, PHD *
PROFESSOR
DEPARTMENT OF HEALTH MANAGEMENT AND INFORMATICS
UNIVERSITY OF MISSOURI SCHOOL OF MEDICINE
COLUMBIA, MO 65212

RODIN, ANDREI, PHD *
SUSUMU OHNO CHAIR IN THEORETICAL BIOLOGY
ASSOCIATE PROFESSOR
DEPARTMENT OF COMPUTATIONAL AND QUANTITATIVE MEDICINE
CITY OF HOPE
DUARTE, CA 91010

SABUNCU, MERT RORY, PHD *
ASSOCIATE PROFESSOR
SCHOOL OF ELECTRICAL AND COMPUTER ENGINEERING
CORNELL UNIVERSITY AND CORNELL TECH
NEW YORK, NY 10044

VALAFAR, FARAMARZ, PHD *
PROFESSOR
SCHOOL OF PUBLIC HEALTH
BIOMEDICAL INFORMATICS RESEARCH CENTER
SAN DIEGO STATE UNIVERSITY
SAN DIEGO, CA 92182

VULLIKANTI, ANIL, PHD *
PROFESSOR
DEPARTMENT OF COMPUTER SCIENCE
AND BIOCOMPLEXITY INSTITUTE
UNIVERSITY OF VIRGINIA
CHARLOTTESVILLE, VA 22904

WANG, DONGMEI, PHD
PROFESSOR
DEPARTMENT OF BIOMEDICAL ENGINEERING
GEORGIA CANCER COALITION
GEORGIA INSTITUTE OF TECHNOLOGY
ATLANTA, GA 30332

WEI, WEI-QI, MD, PHD *
ASSISTANT PROFESSOR
DEPARTMENT OF BIOMEDICAL INFORMATICS
VANDERBILT UNIVERSITY MEDICAL CENTER
NASHVILLE, TN 37203

YETISGEN, MELIHA, PHD *
ASSOCIATE PROFESSOR, BIOMEDICAL AND HEALTH INFORMATICS
BIOMEDICAL AND HEALTH INFORMATICS
UNIVERSITY OF WASHINGTON
SEATTLE, WA 98109

ZHAO, LILI, PHD *
RESEARCH ASSOCIATE PROFESSOR
DEPARTMENT OF BIOSTATISTICS
UNIVERSITY OF MICHIGAN
ANN ARBOR, MI 48109

MAIL REVIEWER(S)

BARTSCH, DIRK-UWE G, PHD
ADJUNCT PROFESSOR
DEPARTMENT OF OPHTHAMOLOGY
SHILEY EYE INSTITUTE
UC SAN DIEGO HEALTH
LA JOLLA, CA 92093

SCIENTIFIC REVIEW OFFICER

SHIVAKUMAR, CHITTARI V, PHD
SCIENTIFIC REVIEW OFFICER
NATIONAL INSTITUTES OF HEALTH
CENTER FOR SCIENTIFIC REVIEW
BETHESDA, MD 20892

EXTRAMURAL SUPPORT ASSISTANT

JONES, BELINDA
EXTRAMURAL SUPPORT ASSISTANT
CENTER FOR SCIENTIFIC REVIEW
NATIONAL INSTITUTES OF HEALTH
BETHESDA, MD 20892

* Temporary Member. For grant applications, temporary members may participate in the entire meeting or may review only selected applications as needed.

Consultants are required to absent themselves from the room during the review of any application if their presence would constitute or appear to constitute a conflict of interest.
